# Patient–physician (dis)agreement on their reports of body surface area affected by psoriasis and its associations with disease burden

**DOI:** 10.1111/jdv.18400

**Published:** 2022-07-14

**Authors:** N. da Silva, M. Augustin, C. Hilbring, C.C. von Stülpnagel, R. Sommer

**Affiliations:** ^1^ German Center for Health Services Research in Dermatology (CVderm) Institute for Health Services Research in Dermatology and Nursing (IVDP), University Medical Center Hamburg‐Eppendorf (UKE) Hamburg Germany

Editor

The people‐centred model of care for psoriasis emphasizes the importance of prioritizing patient's needs and preferences in clinical decisions, along with physician‐rated disease severity.^1^ However, patients and physicians often differ on their perceptions of disease severity,^2^ which might influence treatment adherence/satisfaction and health outcomes. This study aimed at understanding the extent and direction of patient–physician (dis)agreement on their reports of body surface area (BSA) affected by psoriasis and exploring which patient‐reported outcomes (PROs) might contribute to explain the disagreement.

The current analyses used data from the Pruri‐Impact study^3^ and included 110 patients with psoriasis, who completed a sociodemographic/clinical datasheet and standardized PRO questionnaires. A detailed description of patients, methods and measures was published elsewhere.^3^ The physicians reported directly on the total %BSA affected by psoriasis and on the Psoriasis Area and Severity Index (PASI)^4^ categories for head, trunk, arms and legs. The patients' reports of BSA were based on a high‐resolution grid scheme of topology of psoriasis,^5^ which allowed the computation of a total %BSA and %BSA affecting the head, trunk, arms and legs, subsequently categorized according to PASI thresholds. The extent of disagreement was computed from the absolute difference between the patient's and the physician's ratings of total %BSA (¦patient‐report – physician‐report¦) and the direction of disagreement from the difference between the PASI category assigned by the patient and by the physician (patient‐report – physician‐report).

Fair–moderate levels of agreement were found: intraclass correlation coefficient = 0.51 for total %BSA, Cohen's Kappa coefficient = 0.39 for %BSA affecting the head, *k* = 0.36 for trunk, *k* = 0.49 for arms and *k* = 0.41 for legs. There was no significant difference between patients' and physicians' reports of total %BSA (ANOVA for repeated measures: *F* = 0.11, *P* = 0.741), but significant differences between raters for the PASI categories of area affecting the head (Wilcoxon test: *Z* = −2.98, *P* = 0.003), trunk (*Z* = −2.45, *P* = 0.014), arms (*Z* = −2.71, *P* = 0.007) and legs (*Z* = −3.57, *P* < 0.001) were found. The examination of direction of disagreement showed that physicians were more likely to rate %BSA lower than the patients (Fig. 1).

**Figure 1:**
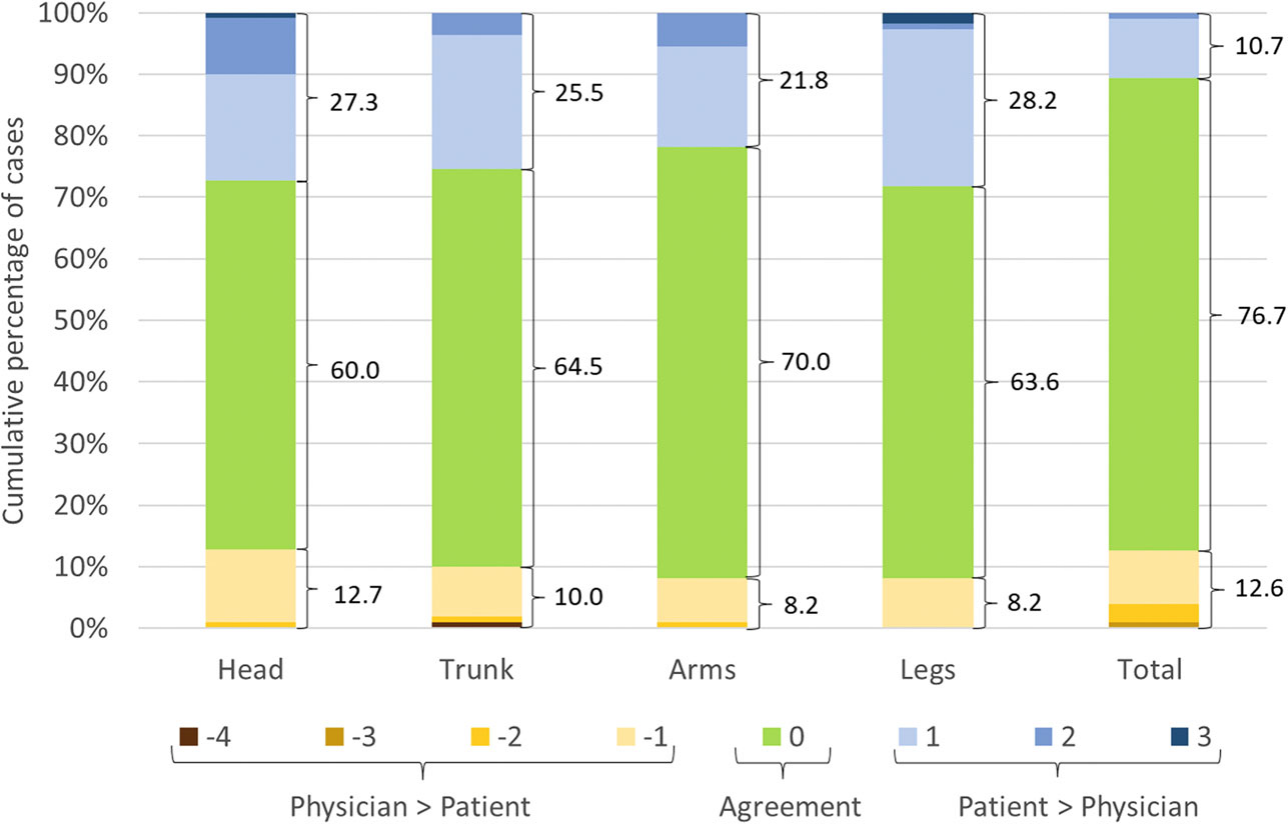
Distribution of the direction of patient–physician disagreement on their reports of body surface area (%BSA) affected by psoriasis in the total body area, and in the head, trunk, arms and legs, categorized according to PASI (0 = none; 1 = <10%; 2 = 10–29%; 3 = 30–49%; 4 = 50–69%; 5 = 70–89%; 6 = 90–100%). The direction of disagreement was computed from the difference between the PASI category assigned by the patient and the PASI category assigned by the physician (patient‐report – physician‐report). Agreement was defined when the patient and the physician assigned a %BSA pertaining to the same category, that is the difference between categories equals to zero.

In addition, a greater extent of disagreement was significantly associated with more quality of life (QoL) impairments, higher levels of depression and lower levels of anxiety (linear regression analysis: *R* ^2^ = 0.32; *F*_(8,66)_ = 3.83, *P* = 0.001; Table 1). The model explaining the direction of disagreement was also significant (multinomial logistic regression; Cox and Snell *R* ^2^ = 0.31, Nagelkerke's *R* ^2^ = 0.44; *χ* ^2^_(16)_ = 28.24, *P* = 0.030; Table 1), but only QoL impairments were significantly associated with patients' likelihood to rate %BSA higher than the physicians.

**Table 1:** Linear regression and multinomial regression analyses explaining the extent and direction of patient‐physician disagreement in total %BSA

Extent of disagreement†	*B* (*SE*)	*t*	*P*	95% CI
Skin‐generic QoL (DLQI)	0.74 (0.27)	2.76	0.007	[0.20, 1.27]
Pruritus‐specific QoL (ItchyQoL)	−1.22 (1.65)	−0.74	0.460	[−4.51, 2.06]
Patient‐defined treatment benefits (PBI)	0.29 (1.16)	0.25	0.804	[−2.03, 2.62]
Depression (PHQ‐2)	7.36 (3.63)	2.03	0.047	[0.11, 14.61]
Anxiety (GAD‐2)	−9.06 (3.82)	−2.37	0.021	[−16.69, −1.42]
Dysmorphic concerns (DCQ)	2.38 (2.42)	0.98	0.329	[−2.45, 7.21]
Perceived stigmatization (PSQ)	2.50 (2.83)	0.89	0.379	[−3.15, 8.15]
Sexual dysfunction (RSS)	0.19 (0.19)	1.00	0.321	[−0.19, 0.56]
**Direction of disagreement: patient < physician (vs. agreement)** ‡	**B (SE)**	**Wald**	***P***	**OR [95% CI]**
Skin‐generic QoL (DLQI)	0.13 (0.11)	1.31	0.252	1.14 [0.91, 1.42]
Pruritus‐specific QoL (ItchyQoL)	−0.67 (0.79)	0.72	0.396	0.51 [0.11, 2.39]
Patient‐defined treatment benefits (PBI)	−0.51 (0.58)	0.78	0.377	0.60 [0.19, 1.86]
Depression (PHQ‐2)	−0.30 (1.45)	0.04	0.836	0.74 [0.04, 12.61]
Anxiety (GAD‐2)	2.22 (1.76)	1.60	0.206	9.24 [0.30, 289.57]
Dysmorphic concerns (DCQ)	−1.69 (0.99)	2.90	0.089	0.19 [0.03, 1.29]
Perceived stigmatization (PSQ)	1.93 (1.49)	1.69	0.194	6.90 [0.38, 127.11]
Sexual dysfunction (RSS)	−0.07 (0.10)	0.51	0.477	0.93 [0.76, 1.13]
**Direction of disagreement: patient > physician (vs. agreement)** ‡	**B (SE)**	**Wald**	***P***	**OR [95% CI]**
Skin‐generic QoL (DLQI)	0.29 (0.13)	4.08	0.043	1.30 [1.01, 1.66]
Pruritus‐specific QoL (ItchyQoL)	0.76 (0.89)	0.73	0.393	2.15 [0.37, 12.38]
Patient‐defined treatment benefits (PBI)	0.81 (0.59)	1.88	0.170	2.25 [0.71, 7.19]
Depression (PHQ‐2)	1.14 (1.70)	0.45	0.503	3.12 [0.11, 86.85]
Anxiety (GAD‐2)	−2.04 (1.74)	1.38	0.241	0.13 [0.004, 3.94]
Dysmorphic concerns (DCQ)	−0.55 (1.11)	0.25	0.620	0.58 [0.07, 5.08]
Perceived stigmatization (PSQ)	−0.35 (1.23)	0.08	0.777	0.71 [0.06, 7.82]
Sexual dysfunction (RSS)	0.03 (0.08)	0.16	0.691	1.03 [0.88, 1.22]

† The extent of disagreement was computed from the difference between the patient's and the physician's ratings of total %BSA affected by psoriasis (continuous variable ranging from 0 to 100), without considering who rated higher (¦patient‐report – physician‐report¦).

‡ The direction of disagreement was computed from the difference between the patient's and the physician's ratings of total %BSA, after they were categorized according to PASI thresholds (patient‐report – physician‐report). Afterwards, the direction of disagreement was categorized into three groups: patient‐report > physician‐report, ‘agreement’ (when the patient and the physician assigned a %BSA pertaining to the same PASI category) and patient’‐report < physician‐report.

B, regression coefficient; CI, confidence interval; DCQ, Dysmorphic Concern Questionnaire (score range from 0 to 21, with scores ≥11 indicating significant concerns in bodily appearance); DLQI, Dermatology Life Quality Index (score range from 0 to 30, with higher scores indicating more QoL impairments); GAD‐2, Generalised Anxiety Disorder (score range from 0 to 6, with scores ≥3 indicating clinically significant symptoms of anxiety); ItchyQoL (score range from 1 to 5, with higher scores indicating more QoL impairments); OR, odds ratio; PBI, Patient Benefit Index (score range from 0 to 4, with higher scores indicating more patient benefits); PHQ‐2, Patient Health Questionnaire (score range from 0 to 6, with scores ≥3 indicating clinically significant symptoms of depression); PSQ, Perceived Stigmatization Questionnaire (score range from 0 to 4, with higher scores indicating higher levels of perceived stigmatization); RSS, Relationship and Sexuality Scale (score range from 0 to 36, with higher scores indicating higher dysfunction); SE, standard error.

The different methods used to assess the %BSA affected by psoriasis are an irrefutable limitation of this study; however, they also raise important considerations: when physicians used a continuous scale, the agreement with the patients' perceptions was higher; when the PASI categories were used, a tendency to rate %BSA lower than the patients was observed. Because all three methods imply subjective perceptions, studies to improve the assessment of psoriasis severity are imperative (e.g. PASI‐HD,^6^ computer‐guided PASI measurement^7^).

Increased QoL impairments were associated with greater disagreement, particularly in the direction of patients rating %BSA higher than physicians. Clinical decisions should therefore take into account the disease burden that even small areas affected, but often visible, can pose to patients' lives. Furthermore, patients' mental health can interfere with effective communication/shared decision‐making (e.g. depressed patients might be more passive/less prone to advocate for themselves; anxious patients might ask more questions/share more concerns as part of their safety behaviour repertoire).^8^, ^9^ Mental health should, therefore, be screened in routine care for psoriasis to improve patient–physician agreement^10^ and facilitate clinical decisions that meet patients' needs and maximize treatment benefits/satisfaction.

## Conflicts of interests

N. da Silva, C. Hilbring and C. C. von Stülpnagel declare no conflict of interest. M. Augustin has served as consultant and/or paid speaker for and/or has received research grants and/or honoraria for consulting and/or scientific lectures for and/or got travel expenses reimbursed and/or participated in clinical trials sponsored by LEO Pharma. R. Sommer has received speaker and/or research honoraria and/or travel expenses by Leo Pharma.

## Funding sources

This study was part of the project ‘Significance of chronic pruritus for social stress and disfigurement in psoriasis – health care study to characterize the need for action and awareness’ (Pruri‐Impact), which was supported by LEO Pharma GmbH.

## Data Availability

The data are available on reasonable request from the corresponding author.
